# Covered endovascular reconstruction of the aortic bifurcation: A versatile technique with many potential applications

**DOI:** 10.1177/17085381241242861

**Published:** 2024-03-28

**Authors:** Christos Lioupis, Luc Francoeur, Vikash Prasad

**Affiliations:** 1 Department of Vascular Surgery, The Moncton City Hospital, 3688 Dalhousie University, Moncton, NB, Canada; 2 Department of Interventional Radiology, The Moncton City Hospital, 3688 Dalhousie University, Moncton, NB, Canada

**Keywords:** Covered Endovascular Reconstruction of the Aortic Bifurcation, seat belt aorta, trauma, aortic occlusion, aorto-enteric fistula

## Abstract

We present our experience with using the bifurcated covered endovascular reconstruction technique in two unusual conditions. One patient with a traumatic “seat belt” aortic injury and a second patient with a bleeding aorto-enteric fistula were successfully treated with the CERAB technique. We discuss potential benefits of the technique outside the usual pattern of chronic atherosclerotic aortoiliac disease.

## Introduction

The Covered Endovascular Reconstruction of the Aortic Bifurcation (CERAB) was introduced to treat complex and extensive aortoiliac occlusive disease.^1^ The technique reconstructs the aortic bifurcation in a more anatomical way, and it seems to be related to superior geometry and more physiologic flow patterns.^2,3^

In this report, we present our experience with using the bifurcated covered endovascular reconstruction technique in two unusual conditions. A patient with a traumatic “seat belt” aortic occlusion was treated successfully with the CERAB technique. A second patient had bifurcated covered endovascular reconstruction of the aortic anastomosis of a previous aorto-bifemoral graft (ABF) for a bleeding aorto-enteric fistula.

We discuss potential benefits of the CERAB technique outside the usual pattern of chronic atherosclerotic aortoiliac disease.

## Case 1

**Figure 1.**
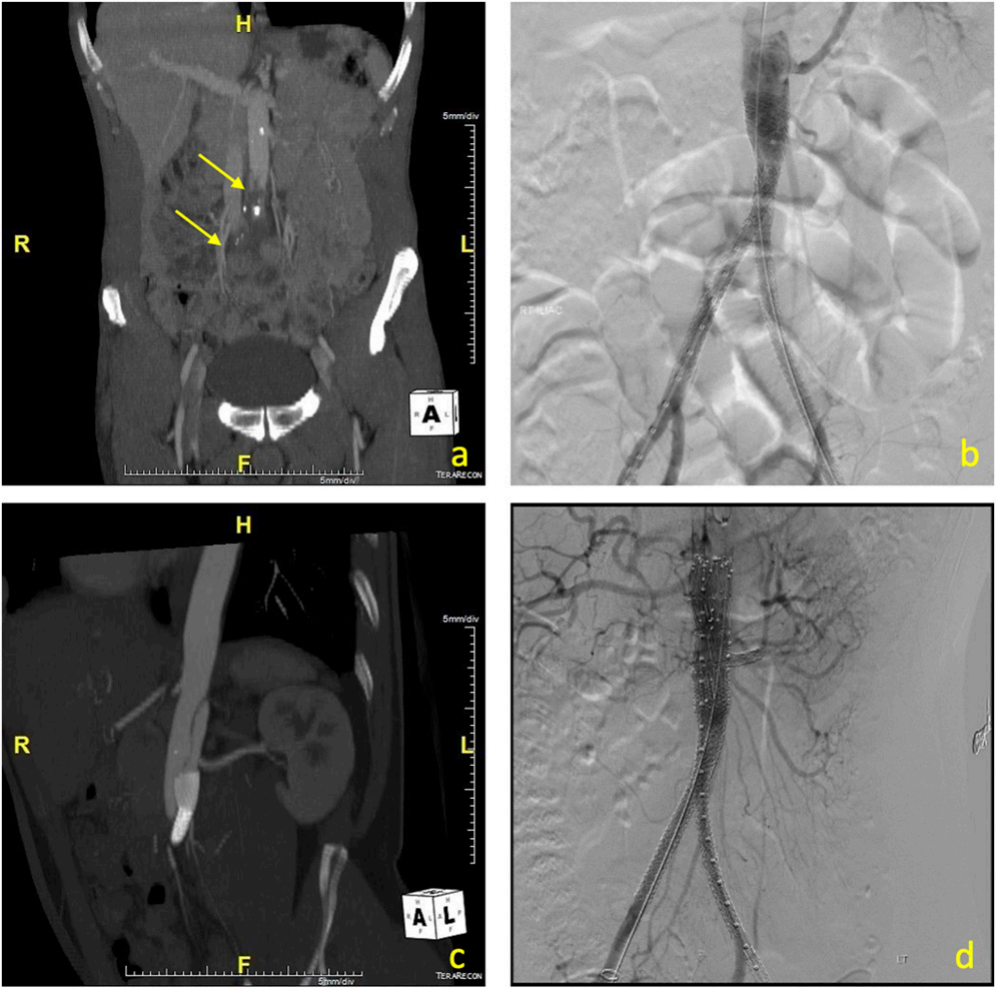
(a) CT angiogram of the abdomen showing occlusion of the aortic bifurcation (yellow arrows), (b) aortoiliac reconstruction with the CERAB procedure, note of quite low origin of left renal artery, (c) follow-up CT angiogram, showing proximal flow limiting dissection of the aorta above the CERAB procedure, (d) proximal extension with custom-made fenestrated cuff with one fenestration for the left renal artery and one scallop for the right renal artery (combined CERAB and fenestrated graft).

A 76-year-old male sustained a “seat belt injury” during a motor vehicle accident. He developed acute bilateral lower limb ischemia (Rutherford IIb) and concomitant small bowel perforation (as documented in a CT angiogram of the abdomen) (Figure 1(a)). Surgical revascularization was not considered to be a good option because of the presence of abdominal bowel contamination, the need for systemic intraoperative anticoagulation, and the time required to re-establish lower-limb perfusion. An endovascular approach was selected instead, with recanalization of the occluded aortoiliac segment and CERAB reconstruction, followed by laparotomy and segmental small bowel resection.

Under general anesthesia, the femoral bifurcations were exposed through two small transverse groin incisions and the origins of the superficial and profunda femoral arteries were shortly clamped during sheath propagation and stent deployment to prevent distal embolization. Recanalization of the aorta required the use of a re-entry device (Outback® Elite catheter, Cordis, Florida, USA). Re-entry was established just below the origin of the left renal artery—that was branching off the aorta in an unusually low position (Figure 1(b)). A 12 × 59 Advanta V12 (Atrium, Hudson, NH, USA) was initially deployed 1 cm above the aortic bifurcation, and it was post-dilated proximally to 16 mm with an Atlas balloon (Bard Peripheral Vascular, Inc., Temple, US). The CERAB procedure was completed with two 8 × 59 Advanta V12 stents deployed in a kissing configuration at the distal third of the proximal aortic covered stent. The left iliac limb was extended to the left iliac bifurcation with a second 8 × 38 Advanta V12 stent. Completion angiogram indicated good patency of the aorta and both iliac arteries. The time required to reperfuse the lower limbs after induction to anesthesia was 45 min. The patient remained hemodynamically stable during the procedure. We used frequent flushing of the sheaths with heparinized solution to prevent thrombus formation, till the aorta had been successfully recanalized and a diagnostic angiogram indicated no acute extravasation from the visceral arteries. The patient then anticoagulated and the heparin was reversed with protamine at the end of the endovascular part of the surgery.

Bilateral four-compartment leg fasciotomies were performed subsequently. After closing the groin incisions, a midline laparotomy followed with segmental small bowel resection. The patient recovered well and regained full mobility of his lower limbs. Antibiotics (Ceftriaxone 2 g 24-hourly + Metronidazole 500 mg 6-hourly) were administered for 3 days postoperatively. Follow-up ultrasound and CT angiogram at 2 weeks post-surgery revealed proximal flow limiting dissection of the aorta, causing dynamic occlusion of the left renal artery ostium (Figure 1(c)), that required a proximal extension using a custom-made fenestrated cuff that was combined successfully with the previous CERAB. The fenestrated cuff (Cook Medical, Bloomington, IN) was designed with one fenestration for the left renal artery and one scallop for the right renal artery, and a conical configuration (22 mm in proximal diameter and 18 mm distally). Three years follow-up is currently available showing good patency of all the stents (Figure 1(d)).

## Case 2

A 72-year-old female patient was transferred to our institute with abdominal pain, hypovolemia, and hematochezia. She had undergone a Dacron ABF graft for occlusive aortoiliac disease two decades ago, with end to side proximal anastomosis. A CT angiogram performed at the local hospital—where she was initially presented—indicated an aorto-enteric fistula with erosion of the aortic graft anastomosis into the adjacent duodenum. We selected to proceed with a staged approach, involving stenting of the proximal anastomosis as a “bridge” to surgical graft excision and aortic reconstruction. Due to the small diameter of the infrarenal aorta (11 mm), no bifurcated aortic stent graft was deemed appropriate to accommodate the anatomy. Perhaps an iliac limb component or a balloon expandable covered stent could have been deployed in the aorta and be extended to one of the ABF graft limbs, with occlusion of the contralateral side. However, this approach would require an additional femoral–femoral crossover bypass in the setting of previous groin surgery—and in an unstable patient. We speculated that a percutaneous CERAB procedure could achieve proximal sealing in a faster and more straightforward way.

**Figure 2.**
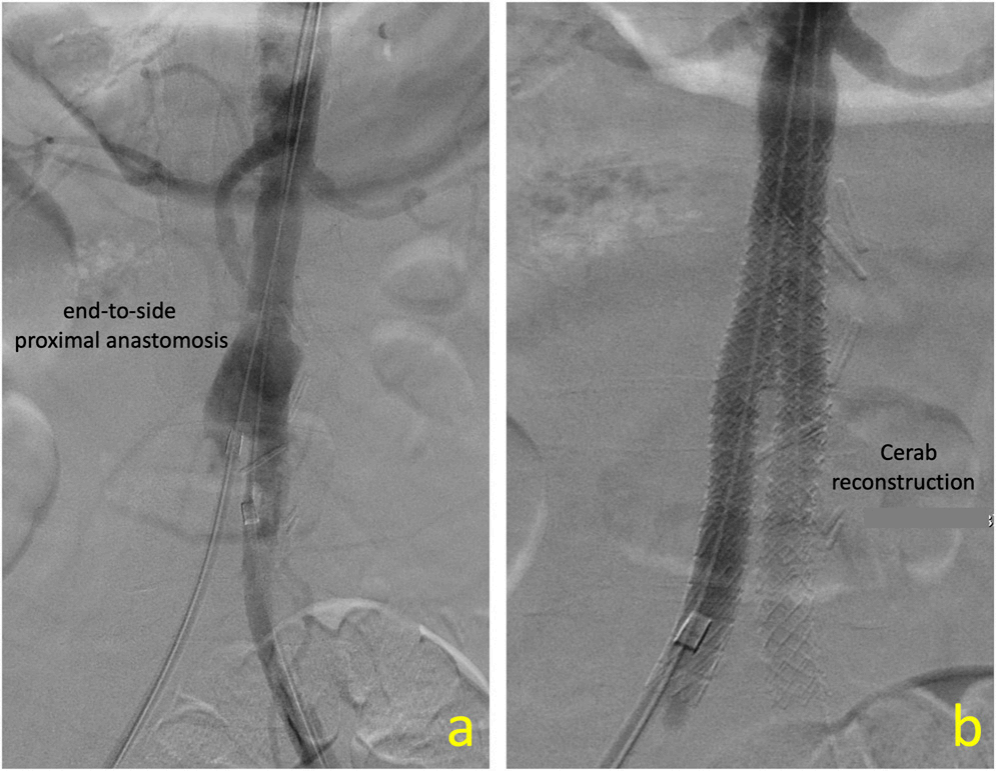
(a) End-to-side proximal anastomosis of ABF graft in a patient of bleeding aorto-enteric fistula. Note the small size of the aorta that was measuring 11 mm and (b) CERAB reconstruction of the aortic anastomosis without any contrast extravasation.

Under general anesthesia, the hoods of the ABF graft femoral anastomoses were cannulated and progressively dilated to allow the advancement of two 8-Fr sheaths inside the limbs of the ABF graft. An 11 × 39 VBX stent graft (Gore Medical, Flagstaff, AZ, USA) was deployed at the junction of the native aorta and the graft anastomosis. We intentionally started 2 cm below the lowest renal artery to allow infrarenal clamping at the second stage procedure of graft extrication. Two 8 × 59 VBX stent grafts were deployed in a kissing configuration at the upper third of the previous stent (higher than our usual approach with standard CERAB procedures). Two additional stents were extended into the limbs of the bifurcated graft and post-dilated at 9 mm distally, due to persisting “Ib type” endoleak. Completion angiogram indicated good sealing of the stent-grafts with no extravasation at the proximal anastomosis (Figure 2). The patient did not systemically heparinized, and we only used frequent flushing of the sheaths with heparinized solution.

The patient was transferred to ICU post-surgery and was resuscitated. She underwent successful extraction of the infected ABF graft 48 h later and creation of a new ABF bypass using an aorto-biiliac RestoreFlow allograft (LeMaitre Vascular, Burlington, MA, USA). The stent-grafts were extracted from the natural aorta without any difficulty. The patient had a successful recovery.

## Discussion

The CERAB technique was developed to manage chronic occlusive disease of the aortic bifurcation. With growing experience, some case reports and small series^4,5^ have demonstrated successful application in different settings. The CERAB reconstruction has been successfully used to reline a chronically occluded bifurcated aortic graft post-EVAR,^4^ or to treat penetrating aortic ulcers located close to the aortic bifurcation, in patients with small diameter iliac arteries.^5^ Recently, we also presented our early experience with using the covered endovascular reconstruction of the iliac bifurcation in patients with non-occlusive aortoiliac disease (penetrating ulcers of the iliac arteries, or even aneurysms involving the internal iliac arteries and/or the iliac bifurcation).^6^

In the two presented cases, the CERAB technique proved to be a fast, simple, and efficient way to reestablish patency of a “seat belt” injured aorta or to seal a leaking aortic anastomosis. The technique has certain advantages. To begin with, it requires use of low-profile sheaths that is particularly important in patients with small size, heavily calcified access arteries or grafts anastomosed to the femoral arteries (second presented case). Furthermore, small size arteries or tapered anatomies can be easily accommodated using balloon expandable covered stents. Moreover, because the limbs of the CERAB configuration are forced into a double-D shape by the tapering part of the proximal balloon-expandable stent grafts there is a lower risk of “gutter-related endoleaks” (provided that the added surfaces of the 2-limb stent-grafts equal the surface of the proximal component). The later proved to be of particular importance in the aorto-enteric fistula case scenario.

Collected experience from the “parallel grafts” technique—that might also be considered an option in the two presented cases—indicates that the use of self-expanding stent-grafts may be associated with endoleaks through the gutters; and this is why a 50-mm overlap is often recommended putting additional anatomic constraints in planning of the procedures.^7^

CERAB is a versatile technique and may be easily combined with other techniques. Preservation of the IMA or renal artery flow can be achieved by combining the CERAB with “chimney grafts.” In the first presented case, a proximal extension was added using a custom-made fenestrated cuff, allowed for complete reconstruction of the abdominal aorta.

## Conclusion

The bifurcated covered endovascular reconstruction may be applied in traumatic aortic injuries to rapidly re-establish patency. The versatility of the technique allows for additional subsequent endovascular interventions when necessary. The use of balloon expandable stent-grafts may also offer an alternative in sealing bifurcated anatomies such as a bleeding aorto-enteric fistula and may be associated with less “gutter-related endoleaks.” Broader experience with the technique may offer more options in management of challenging vascular conditions.
